# Editorial: The expanding functional network of glutathione transferases

**DOI:** 10.3389/fmolb.2023.1146377

**Published:** 2023-01-31

**Authors:** Simona Piaggi, Marc Diederich, Alessandro Corti

**Affiliations:** ^1^ Department of Translational Research and New Technologies in Medicine and Surgery, University of Pisa Medical School, Pisa, Italy; ^2^ College of Pharmacy, Seoul National University, Seoul, South Korea

**Keywords:** glutathione transferases, inflammation, pathogenesis, polymorphisms, steroid biochemistry

The superfamily of glutathione transferases (GSTs) comprises several distinct and multifunctional proteins widely distributed in Nature among eukaryotes and prokaryotes. GSTs are divided according to their cellular localization, with the cytosolic GSTs largely distributed in eukaryotes and further subgrouped into seven distinct classes of enzymes (Mannervik et al., 2005; Board and Menon, 2013).

Besides the early recognized involvement of GSTs in the metabolism of drugs and xenobiotics, a growing number of data has revealed other biological functions of GSTs. These include isomerase reactions, protein glutathionylation, and the regulation of signaling pathways (Board and Menon, 2013). Non-enzymatic functions, including the regulation of the MAPK pathway (Adler et al., 1999; Pljesa-Ercegovac et al., 2018) and ryanodine receptors (Dulhunty et al., 2011), have also been proposed for some GSTs. During recent years, the interest in GSTs has increased due to their involvement in varied fields of biology and medicine. Moreover, the development of specific GST inhibitors has become an active topic of study.

The reviews and research articles of the present Research Topic represent an attempt to focus on the novel, not yet fully explored mechanisms and functions of GSTs.

Mannervik and colleagues review the ketosteroid isomerase activity of GSTs in the first manuscript of this research topic. The authors summarize and discuss studies about the contribution of alpha-class GSTs (GSTA) to steroid hormone biosynthesis in mammalian tissues. Human GSTA3-3 and GSTA1-1 isoforms support the activity of the 3β-hydroxysteroid dehydrogenase, which catalyzes the steroid isomerization to produce progesterone and 4-androstenedione from their delta 5-precursors. GSTA3-3 plays the highest ketosteroid isomerase activity in human and equine steroidogenic tissues. The mechanism involving the thiolate of glutathione (GSH), the mammalian orthologs of GSTA3-3, the available structural analysis, and the substrate selectivity are discussed. Altogether, this manuscript exemplifies how GSTs acquired novel functions in steroid biochemistry.

The following two papers are dedicated to GST polymorphisms and their significance in lung inflammation. This is a very active field of research with many possible pathophysiological implications related to the different functions of GSTs. Besides the classical reactions of detoxification catalyzed by GSTs against harmful environmental compounds, their regulatory functions on intracellular inflammatory signaling are considered here (Menon et al., 2015; Menon et al., 2017; Hughes et al., 2019). GST polymorphisms were also correlated with the increased risk of developing chronic inflammatory lung diseases (Ishii et al., 1999; Yanbaeva et al., 2009; Piacentini et al., 2013; Piaggi et al., 2021; van de Wetering et al., 2021). The review by Dai and colleagues summarizes recent data about GSTP1, GSTM1, and GSTT1 polymorphisms, the three most studied classes of GST. The authors focused on their connection with household air pollution exposure and their possible role in the etiology of asthma. GST polymorphisms are discussed in the context of interactions with antioxidants and with possible sources of oxidative stress in the lung. Differential antioxidant/detoxifying capacity or regulatory functions may help understand the multiple functions of GST polymorphisms and unveil susceptibility profiles in the general population. In this perspective, the original research paper by Coric and colleagues focuses on the significance of cytosolic GSTA1, GSTM1, GSTM3, GSTP1, and GSTT1 polymorphisms in the severe acute respiratory syndrome Coronavirus (SARS-CoV)-2 infection. Undoubtedly, a clearer picture of correlations and pathogenetic mechanisms of GST polymorphisms in lung inflammation could support the development of innovative therapeutic approaches.

The role of GSTs also appears essential in the field of neuroinflammation. The central nervous system is highly sensitive to toxins and oxidative stress due to low antioxidant enzyme levels, high content of prooxidants, and ROS accumulation. There is a general agreement about the protective effect of GSH and GSTs. Still, the mechanisms underlying this crucial protective role have not yet been defined in neurodegenerative diseases (Mazzetti et al., 2015). However, several GST polymorphisms were found to correlate with the onset of neurodegenerative conditions, eventually leading to Alzheimer’s and Parkinson’s diseases (Allocati et al., 2018). GSTM1 is the major brain isoform expressed in neurons and astrocytes (Mazzetti et al., 2015). The brief research report by Matoba and colleagues investigates the role of GSTM1 after pro-inflammatory stimulation of astrocytes. The effects of reduced GSTM1 expression levels on the TNF-α-dependent transcriptome and LPS-induced responses are investigated. The authors extend this study to GSTM1 expression in aging mice’s brains. A deeper understanding of the roles of GSTs in the pathogenesis of brain inflammation could improve the identification of novel therapeutic targets and the development of new and specific drugs.

The paper by Sylvestre-Gonon and colleagues documents the wide distribution of GST expression and their alternative functions in Nature. Indeed, this original research paper focuses on the Tau class of GSTs (GSTU) and its GSTU19 and 20 paralogs in *Populus trichocarpa* (poplar tree). Besides the more classical GSH-conjugation and peroxidase activities, GSTU paralogs would also play a role in the metabolism or trafficking of flavonoids. This result highlights another atypical function of GSTs. These enzymes can bind and transport a wide range of small heterocyclic ligands by their non-catalytic ligandin properties (Mannervik and Danielson, 1988; Brock et al., 2013).

In summary, the articles collected in the present Research Topic represent a variety of updated data and hypotheses that reflect the interdisciplinarity of the GST superfamily. This result was somewhat expected due to the wide distribution of the multifunctional GSTs in Nature. Collections of papers of this kind can usefully open novel perspectives about the physiopathological functions of structurally and functionally similar enzymes expressed by different tissues and–even more interestingly–by very different living organisms. The understanding of the functions of human and non-human GSTs will eventually lead to a better understanding of the yet unrecognized roles of GSTs in human diseases and the development of suitable pharmacological interventions.
